# Treating Patients with Aripiprazol: A Safe Gamble?

**DOI:** 10.1192/j.eurpsy.2022.1838

**Published:** 2022-09-01

**Authors:** B. Leal, D. Vila-Chã, S. Garcia, I. Pinto, R. Mateiro, M. Avelino, M. Martins, J. Salgado

**Affiliations:** Centro Hospitalar Psiquiátrico de Lisboa, Clínica 1, Lisboa, Portugal

**Keywords:** Gambling Disorder, Aripiprazol, Dopamine receptor

## Abstract

**Introduction:**

Aripiprazole (ARI) is an atypical antipsychotic drug with D2 partial agonist properties, usually prescribed to treat mood disorders (major depression or bipolar disorder) and schizophrenic disorder (schizophrenia or schizoaffective disorder). Dopamine receptor agonists, as is ARI, have been implicated in some cases of impulse-control problems, such as gambling disorder (GD), increased spending, hypersexuality and compulsive eating.

**Objectives:**

Currently, it is hypothesized that aripiprazole may cause impulse-control problems because it can produce a hyperdopaminergic state in the mesolimbic pathway (reward system) through its predominant action on dopamine D3 receptors. We intend to do a non-systematic review of the scientific information regarding this subject.

**Methods:**

The authors revised the published literature about this topic, selecting relevant articles, systematic reviews and case reports, with the topic words: “aripiprazol”, “gambling disorder” and “dopamine receptor” in scientific data base.

**Results:**

Overall, a few cases of ARI-induced pathological gambling as well as ARI-induced hypersexuality have been reported. In one study it was verified that comorbid psychiatric and substance use disorders were common among those who have experienced GD or worsened GD after beginning ARI treatment. In another study, it was verified that the group of patients who reported this alleged side-effect were mostly young (mean age, 33.6 years), mostly men (88.2%) and most lived alone.

**Conclusions:**

Attributing to dopamine agonists the only factor that can explain the onset of GD is simplistic and dangerous. Many other potential risk factors, including individual vulnerability factors (temperament, genetics) as well as environmental factors, must be considered.

**Disclosure:**

No significant relationships.

